# Antinociceptive and anti-inflammatory activities of the ethanolic extract of *Annona vepretorum* Mart. (*Annonaceae*) in rodents

**DOI:** 10.1186/s12906-015-0716-2

**Published:** 2015-06-24

**Authors:** Juliane C Silva, Camila de S Araújo, Sarah Raquel G de Lima-Saraiva, Raimundo G de Oliveira-Junior, Tâmara C Diniz, Carlos Wagner de S Wanderley, Raimundo C Palheta-Júnior, Rosemairy L Mendes, Adriana G Guimarães, Lucindo J Quintans-Júnior, Jackson Roberto G da S Almeida

**Affiliations:** Center for Studies and Research of Medicinal Plants, Federal University of San Francisco Valley, 56.304-205 Petrolina, Pernambuco Brazil; Department of Physiology, Federal University of Sergipe (DFS/UFS), 49.100-000 São Cristóvão, Sergipe Brazil

**Keywords:** Pain, Inflammation, *Annona vepretorum*

## Abstract

**Background:**

*Annona vepretorum* Mart. (Annonaceae) is a native tree from Caatinga (Brazilian Northeastern savanna biome), popularly known as “araticum” and “pinha da Caatinga”. In this study, we investigated the effects of the crude ethanolic extract (Av-EtOH) in models of pain and inflammation in rodents.

**Methods:**

The evaluation of antinociceptive activity was carried out by the acetic acid-induced writhing, formalin, hot plate and tail flick tests, while paw edema induced by carrageenan or histamine, and leukocyte migration to the peritoneal cavity were used for anti-inflammatory profile. Histological analyses also were carried out.

**Results:**

Av-EtOH (25, 50 and 100 mg/kg, p.o) significantly reduced the number of writhing (*P* < 0.01) and decreased (*P* < 0.01) the paw licking time in both phases of the formalin test. In the hot plate and tail flick tests, this extract increased the reaction time, consequently reduced painful behavior. The effects in the formalin and hot plate tests were antagonized by naloxone. Av-EtOH inhibited significantly (*P* < 0.01) the increase in the edema volume after administration of carrageenan and histamine. In the peritonitis test, acute pre-treatment with Av-EtOH inhibited leukocyte migration. Histological analysis showed less inflammation in the groups treated with the extract when the inflammation was induced by carrageenan or histamine.

**Conclusion:**

Thus, Av-EtOH has significant antinociceptive and anti-inflammatory properties, which are related probably with the activation of opioid receptors and inhibition of release of mediators of the inflammatory process. This specie is a potential target for drug discovery.

## Background

The Caatinga biome (semi-arid vegetation) is a highly threatened biome covering a vast area in Northeastern Brazil and is the source of few studied natural resources [1]. Many medicinal plants species from Caatinga are widely known and used in folk medicine and for commercial manufacturing of phytotherapeutic products. Few ethnobotanical and pharmacological studies have been undertaken in this region, in spite of the great cultural and biological diversity to be found there [2]. In the Brazilian Northeastern, the Caatinga has fundamental importance in the lives of people that inhabit this region because it offers a wide variety of animals and plants that are used for food, fuel, building materials and medicinal purposes [3].

Plants of the Annonaceae family can be found in tropical and subtropical regions [4]. It is the largest family of the Magnoliales order, having about 2300–2500 species and over 130 genera [5]. Many species of Annonaceae are usually consumed as fresh fruits, they are also widely used in folk medicine as antiparasitic, antitumoral and for treatment of intestinal diseases [6, 7]. Several reports have characterized the pharmacological activity of these plants because of their bioactive compounds (mainly alkaloids and flavonoids) found in roots, leaves, bark, seeds and fruits [5].

*Annona* L. belongs to the Annonaceae family and comprises approximately 175 species, including trees and shrubs. Economically, this genus is the most important of the Annonaceae family due to its edible fruits and medicinal properties [8]. Some studies have demonstrated that species of the genus *Annona* have pharmacological properties mainly antinociceptive [5, 9] and anti-inflammatory activities [10].

*Annona vepretorum* is endemic of Brazil (Caatinga biome), popularly known as “araticum” and “pinha da Caatinga” and is widely used in the human nutrition. The fruits are usually consumed “in natura” or used in juices, and the leaves (decoction) are used in bath to allergies, skin diseases, yeast and bacteria infection, while its roots are indicated to bite of bees and snakes, inflammatory and pains conditions. Previous study of this species described the chemical composition and bioactivity of the essential oil from the leaves collected in Poço Redondo, state of Sergipe, Northeastern Brazil. The major compounds identified were: bicyclogermacrene, spathulenol, α-phellandrene, (*E*)-β-ocimene, α-pinene, *o*-cymene and germacrene D and the essential oil of *A. vepretorum* showed potent cytotoxic activity against eight tumor cell lines out of ten evaluated indicating its promising source of biologically active compounds with cytotoxic properties and stimulate your research *in vivo* assays [11, 12]. Recently, our research group evaluated the central nervous system effects of the ethanolic extract in mice and demonstrated that this plant has sedative activity but does not affect the motor coordination of animals on the rota rod test. The preliminary analysis demonstrated that the ethanolic extract was positive for the presence of phenols, steroids, terpenoids and flavonoids [13].

Considering the large consumption of this species in our region, this study evaluated the antinociceptive and anti-inflammatory properties of the ethanolic extract from *Annona vepretorum* in experimental models of pain and inflammation.

## Methods

### Plant material

The leaves of *A. vepretorum* were collected in Jaguarari, Bahia, Brazil, in October of 2010. The plant was identified by R. Mello Silva, a botanist from Centro de Referência para Recuperação de Áreas Degradadas (CRAD). The voucher specimen (#946) was deposited at the Herbário Vale do São Francisco (HVASF) from Universidade Federal do Vale do São Francisco.

### Preparation of the plant extract

The leaves of *A. vepretorum* were dried in an oven at 40 °C for three days. The material dried and pulverized (400 g) was macerated with ethanol (EtOH) 95 % at room temperature for 72 h. The EtOH solution was concentrated under vacuum yielding 42 g (10.50 % w/w from dried plant material) of crude ethanol extract of *Annona vepretorum* (Av-EtOH).

### Animals

Adult male Swiss mice (30–40 g) and rats (200–250 g), were randomly housed in appropriate cages at 22 ± 2 °C on a 12 h light/dark cycle (lights on at 6:00 a.m.) with access to food and water *ad libitum*. Animals were allowed to have a period of acclimation before any experimentation. They were used in groups of six animals each. All nociception tests were carried out by the same visual observer. Experimental protocols and procedures were approved by the Federal University of San Francisco Valley Animal Care and Use Committee by number 0004/261011.

### Pharmacological tests

#### Acetic acid-induced writhing in mice

This test was performed using the method described by Collier *et al.* [14] with modifications. Mice were divided into six groups of six animals each. Nociception was induced by intraperitoneal (i.p.) injection of acetic acid (0.9 % v/v) in a volume of 0.1 ml/10 g. Animals were treated with the Av-EtOH (25, 50 and 100 mg/kg, p.o.) [13], or with saline (p.o., negative control) 1 h before the nociceptive agent. Indomethacin (20 mg/kg, i.p.) and morphine (10 mg/kg, i.p.) in saline were used as reference drugs and given by i.p. 30 min before algogen agent. Following the injection of acetic acid, the number of abdominal constrictions (a response consisting of contraction of the abdominal wall, pelvic rotation followed by hind limb extension) occurring between 5 and 15 min after injection was counted [15].

### Formalin test

The method used was similar to that described by Hunskaar and Hole [16]. A formalin solution (2.5 % in 0.9 % sterile saline; 20 μl/paw subplantar) was injected into the right hind paw of the mice [17]. Mice were observed in the chambers with a mirror and the amount of time (in seconds) spent licking and biting the injected paw was measured as an indicator of pain. Responses were measured for 5 min after formalin injection (first phase, neurogenic) and 15–30 min after formalin injection (second phase, inflammatory) [18]. Treatments with saline (p.o.), Av-EtOH (25, 50 and 100 mg/kg, p.o.), indomethacin (20 mg/kg, i.p.) and morphine (10 mg/kg, i.p.) were given 1 h prior to formalin injection (*n* = 6 per group). For evaluation of the involvement of opioid receptors in the effect of the extract, naloxone (1.5 mg/kg, i.p.), an opioid receptor antagonist, was administered 30 min before administration of the extract (100 mg/kg) or morphine.

### Hot plate test

Mice were divided into six groups of six mice each. Mice were pre-selected on the hot plate apparatus (Insight, Brazil) at 55 ± 0.5 °C. Animals showing a reaction time (defined as the latency for licking the hind feet or jumping) greater than 20 s were discarded. Selected mice were pre-treated with saline (p.o.), Av-EtOH (25, 50 and 100 mg/kg, p.o.), or morphine (10 mg/kg, i.p.). Each animal was placed on the heated surface of the plate maintained at 55 °C and the latency to a discomfort reaction (licking of the paws or jumping) was recorded at 30, 60, 90 and 120 min after the administration of the saline, extract and morphine [19]. A cut-off time of 20 s was chosen to indicate complete analgesia and to avoid tissue injury. The latencies for paw licking or jumping were recorded for each animal. For evaluation of the involvement of opioid receptors in the effect of the extract, naloxone (1.5 mg/kg, i.p.) was administered 30 min before administration of the extract (100 mg/kg) and morphine.

### Tail flick test

The antinociceptive response against thermal stimuli was assessed with the tail-flick test (Analgesiometer, Insight, Brazil) [20]. Rats were divided into five groups of six rats each and each rat was placed in a ventilated tube with the tail positioned in radiant thermal stimulation of the dorsal surface of the tail. The heating was applied to distal third of the tail and consist focus a beam of light at 50 °C in the tail of rats. The time, in seconds, taken to remove the tail the beam is regarded as the reaction time, which is measured automatically by a recorder coupled to the apparatus. Each trial was terminated after 7 s to minimize the probability of skin damage. The tail-flick latency was measured before and 30, 60, 90 and 120 min after administration of saline (p.o.), Av-EtOH (25, 50 and 100 mg/kg, p.o.), or morphine (10 mg/kg, i.p.) [21].

### Carrageenan-induced hind paw edema in mice

The anti-inflammatory activity was studied using the paw edema model induced by 2 % carrageenan, injected at volume of 20 μl/animal into the subplantar region of the right hind paw of the mice [22]. Mice were divided into six groups of six animals each. Mice were pre-treated with Av-EtOH (25, 50 and 100 mg/kg, p.o.), saline (p.o.) or indomethacin (20 mg/kg, i.p.) 1 h before carrageenan injection or saline injection. The mice pedal volume up to the ankle joint was measured using plethysmometer (PanLab LE 7500, Spain) before (VA, baseline) the intraplantar administration of carrageenan and 1, 2, 3, 4 and 5 h after (VB), as described previously [23]. The inhibition of the edema paw was calculated by (VB-VA)/VA, where VA is the volume of the right hind paw before carrageenan injection, and VB is the volume of the right hind paw after carrageenan injection. Finally, the animals were euthanized and all of right hind paw were dissected. Each sample was embedded in paraffin wax, sectioned at 5 μm and stained with hematoxylin-eosin for histological analysis.

### Histamine-induced hind paw edema in mice

The edema in mice was induced by injecting 100 μg/paw of histamine [24]. Mice were divided into three groups of six animals each. Animals were pre-treated with Av-EtOH (100 mg/kg, p.o.), saline (p.o.) 1 h before histamine injection or saline. The volume was measured before the intraplantar injection of histamine or saline (VA, baseline) and 30, 60, 90 and 120 min after (VB) [25]. The inhibition of paw edema was calculated as described in carrageenan induced paw test. The animals were also sacrificed and all of right hind paw were dissected for histological analysis.

### Leukocyte migration to the peritoneal cavity

Mice were divided into five groups of six animals each. The leukocyte migration was induced by injection of carrageenan (1 %, i.p., 0.25 ml) [26] into the peritoneal cavity of mice 1 h after administration of Av-EtOH (25, 50 and 100 mg/kg, p.o.), or with saline (p.o.) 0.5 h after injection of dexamethasone (2 mg/kg, i.p.) [27]. The leukocyte migration was evaluated 4 h after stimulus, when the animals were euthanized and the peritoneal cavity cells were harvested with 3 ml saline containing 1 mM EDTA. Immediately, a brief massage was done for further fluid collection, which was centrifuged (3000 rpm for 6 min) at room temperature. The supernatant was disposed and saline was added to the precipitate. An aliquot of 10 μl from this suspension was dissolved in 200 μl of Turk solution and the total cells were counted in a Neubauer chamber, under optic microscopy. The results were expressed as the number of leukocytes/ml [28].

### Statistical analysis

The results were presented as the mean ± standard error of the mean (SEM) and the statistical significance was determined using an analysis of variance (ANOVA) followed by Dunnett’s test. Groups of data of histamine-induced hind paw edema in mice were analyzed using unpaired Student’s *t*-test. Values were considered significantly different at *P* < 0.05. All analysis was performed using by GraphPad Prism 5.0 program (Graph Pad Prism Software Inc., San Diego, CA, USA).

## Results

### Acetic acid-induced writhing in mice

**Fig. 1:**
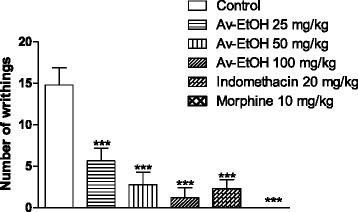
Effect of ethanol extract of *A. vepretorum* (Av-EtOH), indomethacin and morphine on acetic acid induced writhing test in mice. Values are mean ± S.E.M. **P < 0.01, significantly different from control; ANOVA followed Dunnett’s test (*n* = 6, per group)

The results shown in Fig. 1. demonstrated that the pre-treatment with Av-EtOH (25, 50 and 100 mg/kg, p.o.) 1 h before, was capable of inhibiting the abdominal writhing induced by the intraperitoneal administration of the acetic acid when compared with control group (*P* < 0.01). The inhibitions of writhes in percentage were 62, 81 and 92, respectively. Indomethacin and morphine produced 93 and 100 %, respectively, of reduction in acetic acid-induced writhing when compared to the control.

### Formalin test

**Fig. 2:**
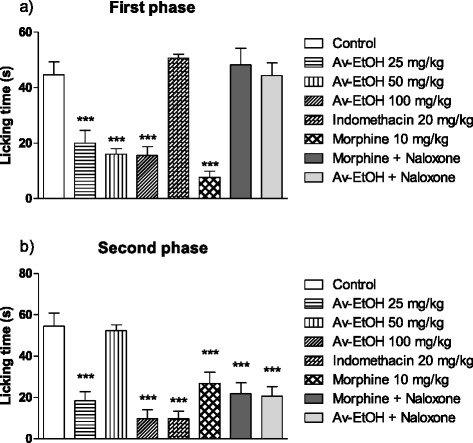
Effect of ethanolic extract of *A. vepretorum* (Av-EtOH), indomethacin, morphine, morphine + naloxone (Morph + NLX; 10 mg + 1.5 mg/kg) and Av-EtOH + NLX (100 mg + 1.5 mg/kg) on formalin test in mice. Values are mean ± S.E.M.; **P < 0.01, significantly different from control; ANOVA followed Dunnett’s test (*n* = 6, per group)

The treatment with Av-EtOH at doses of 25 and 100 mg/kg (p.o.) produced a significant antinociceptive activity compared to the control group in both the early and late phases (*P* < 0.05) (Fig. 2). Av-EtOH (25, 50 and 100 mg/kg, p.o.) decreased by 55, 64 and 65 %, respectively, the paw licking time in the first phase, as well as 66, 4 and 82 %, respectively, in the second phase of the formalin test. The reference drug indomethacin suppressed only the second phase of the formalin test, while morphine inhibited both phases of the pain stimulus (*P* < 0.05). The pre-treatment with naloxone (1.5 mg/kg, i.p.) reversed the antinociceptive activity of the extract at dose of 100 mg/kg in the first phase of this test. The effect of morphine (10 mg/kg) was also reversed by naloxone.

### Hot plate test

**Fig. 3:**
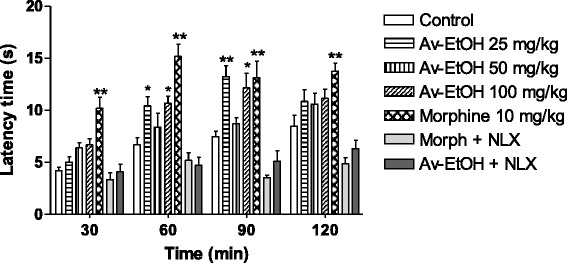
Effect of ethanolic extract of *A. vepretorum* (Av-EtOH), morphine, morphine + naloxone (Morph + NLX; 10 mg + 1.5 mg/kg) and Av-EtOH + NLX (100 mg + 1.5 mg/kg) on hot plate test in mice. Values are mean ± S.E.M.; *P < 0.05, **P < 0.01, significantly different from control; ANOVA followed Dunnett’s test (*n* = 6, per group)

In the hot plate test (Fig. 3), the animals treated with morphine (10 mg/kg, i.p.) demonstrated a marked increase in latency at 30, 60, 90 and 120 min. The ones treated with the Av-EtOH (25 and 100 mg/kg, p.o.) showed a marked increase in latency at 60 and 90 min. The one treated with Av-EtOH (50 mg/kg, p.o.) did not shown significantly behavioral changes. The effect of morphine and Av-EtOH was reverted by naloxone.

### Tail flick test

**Fig. 4:**
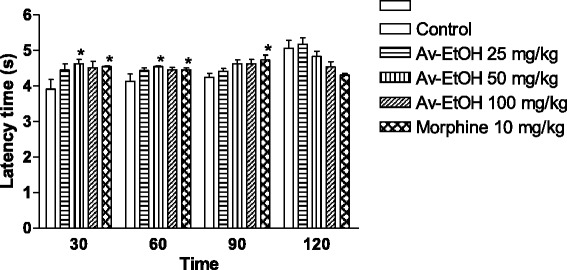
Effect of ethanolic extract of *A. vepretorum* (Av-EtOH) and morphine on tail flick test in rats. Values are mean ± S.E.M.; *P < 0.05, significantly different from control; ANOVA followed Dunnett’s test (*n* = 6, per group)

The Av-EtOH administrated at dose of 50 mg/kg (p.o) induced a significant increase in the reaction time to thermal stimuli when compared to the control group at 30 and 60 min. The group treated with morphine (10 mg/kg, i.p.) caused a significant increase in the response latency time at 30, 60 and 90 min (Fig. 4).

### Carrageenan-induced hind paw edema in mice

**Fig. 5:**
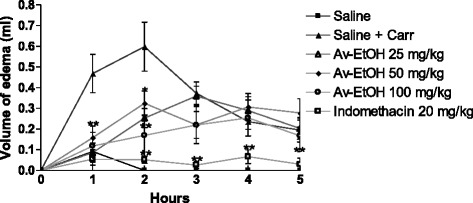
Effect of ethanolic extract of *A. vepretorum* (Av-EtOH) and indomethacin on carrageenan-induced hind paw edema in mice. Values are mean ± S.E.M.; *P < 0.05, **P < 0.01, significantly different from control; ANOVA followed Dunnett’s test (*n* = 6, per group)

When carrageenan was used as inductor of edema (Fig. 5), Av-EtOH (25, 50 and 100 mg/kg, p.o) administered 1 h before carrageenan injection, inhibited significantly (*P* < 0.01) the increase in the edema volume only at 1 and 2 h. Moreover, control drug, indomethacin (20 mg/kg, i.p.), showed the inhibition of edema volume (*P* < 0.01) during 5 h (Table 1).

**Table 1:** Effect of Av-EtOH on carrageenan-induced hind paw edema in mice

Drug treatments	Dose (mg/kg)	Paw volume (ml)				
		1 h	2 h	3 h	4 h	5 h
Saline		0.09 ± 0.01	0.00 ± 0.00	0.00 ± 0.00	0.00 ± 0.00	0.00 ± 0.00
Saline + Carr		0.47 ± 0.09	0.60 ± 0.12	0.37 ± 0.05	0.24 ± 0.04	0.19 ± 0.06
Av-EtOH	25	0.09 ± 0.05***	0.25 ± 0.05**	0.36 ± 0.05	0.29 ± 0.07	0.20 ± 0.05
Av-EtOH	50	0.16 ± 0.06**	0.33 ± 0.06*	0.22 ± 0.06	0.31 ± 0.06	0.28 ± 0.07
Av-EtOH	100	0.12 ± 0.07***	0.17 ± 0.09***	0.22 ± 0.09	0.25 ± 0.09	0.17 ± 0.08
Indomethacin	20	0.06 ± 0.03***	0.05 ± 0.02***	0.03 ± 0.02***	0.07 ± 0.03	0.03 ± 0.03

*P < 0.05; **P < 0.01; ***P < 0.001. Carr: carrageenan

### Histamine-induced hind paw edema in mice

**Fig. 6:**
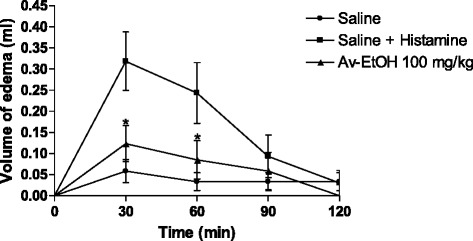
Effect of ethanolic extract of *A. vepretorum* (Av-EtOH) on histamine-induced hind paw edema in mice. Values are mean ± S.E.M.; *P < 0.05, significantly different from control; unpaired Student’s *t*-test (*n* = 6, per group)

The inflammatory edema induced by histamine was significantly attenuated by Av-EtOH 100 mg/kg, p.o. in 30 min and 60 min (*P* < 0.05), as shown in Fig. 6.

### Histological examination

**Fig. 7:**
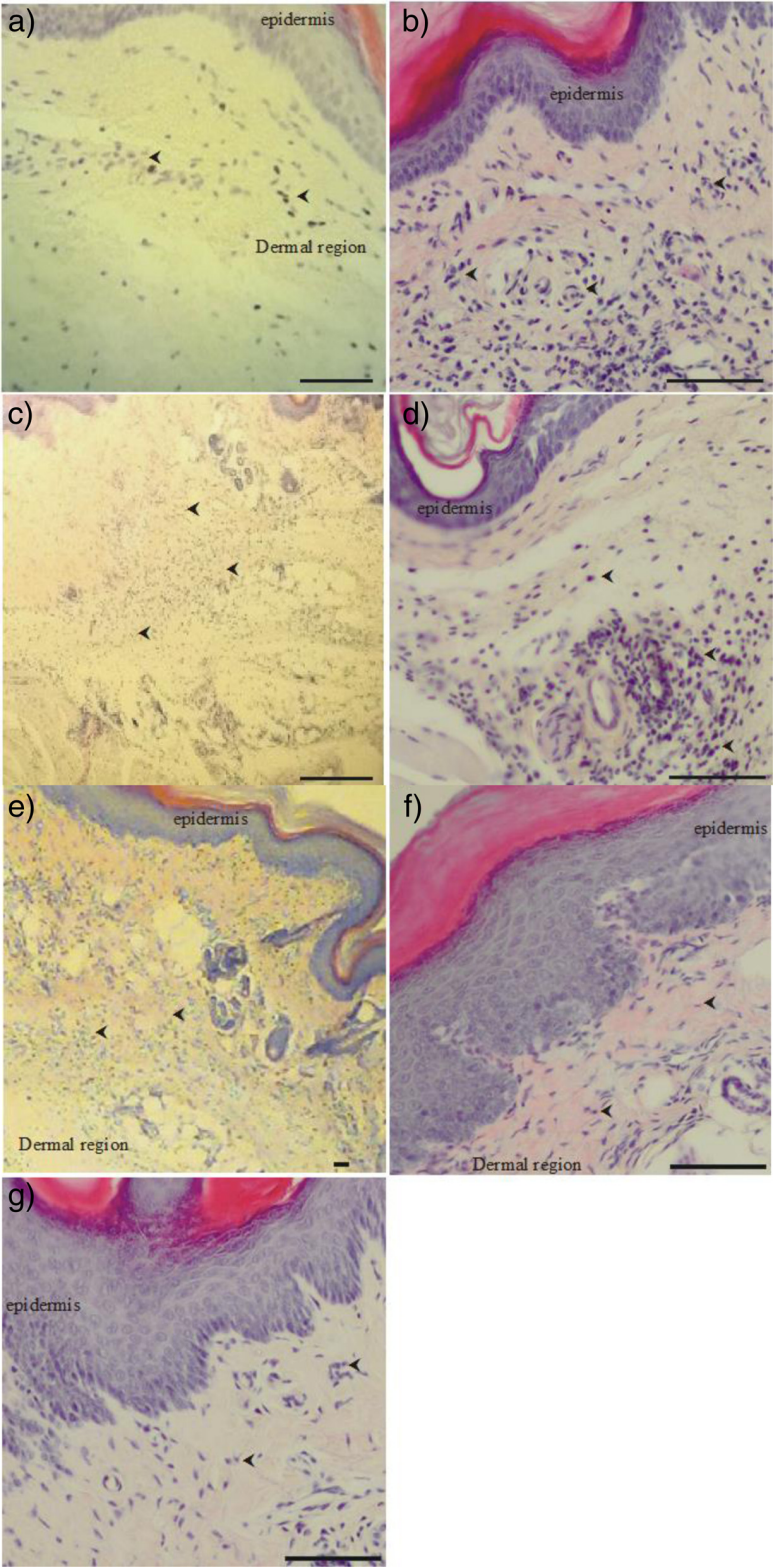
Histology of mice paw after edema induced by carrageenan after treatments. **a** control group (saline + carrageenan), **b** Av-EtOH 25 mg/kg + carrageenan, **c** Av-EtOH 50 mg/kg + carrageenan, **d** Av-EtOH 100 mg/kg + carrageenan, **e** Indomethacin + carrageenan, **f** Histamine + saline, **g** Histamine + Av-EtOH 100 mg/kg.) The arrows head shows neutrophil infiltration of paw tissues (Scale bars: 200 μm (**a**, **c** and **e**) and 50 μm (**b**, **d**, **f** and **g**)

Histological analysis showed the presence of intense inflammatory infiltrate with subcutaneous edema in the groups where inflammation was induced by carrageenan and treated with 50 and 100 mg/kg of Av-EtOH. However, the group treated with 25 mg/kg of Av-EtOH the infiltrate was less intense. In the group where inflammation was induced by histamine and treated with 100 mg/kg of Av-EtOH the inflammatory process was less intense than in the control group (Fig. 7).

### Leukocyte migration to the peritoneal cavity

**Fig. 8:**
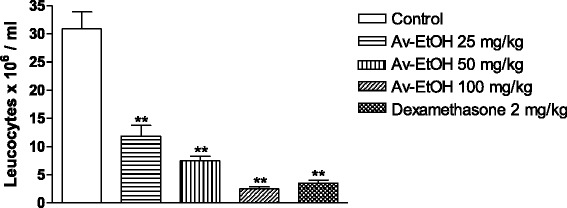
Effect of ethanolic extract of *A. vepretorum* (Av-EtOH) and dexamethasone on leukocytes migration into the peritoneal cavity induced by carrageenan in mice. Values are mean ± S.E.M.; **P < 0.01, significantly different from control; ANOVA followed Dunnett’s test (*n* = 6, per group)

Av-EtOH (25, 50 and 100 mg/kg, p.o) administered 1 h before injection of carrageenan (1 %, i.p., 0.25 ml) inhibited leukocyte migration (*P* < 0.001) when compared to control group (Fig. 8). The reference drug dexamethasone (2 mg/kg, i.p.) promoted significant reduction in leukocyte migration (*P* < 0.001). Thus, the inhibitory effect of Av-EtOH on leukocyte migration to the peritoneal cavity was 62, 76 and 98 % at dose of 25, 50 and 100 mg/kg of Av-EtOH, while 89 % at dose of dexamethasone.

## Discussion

Preliminary analysis of the crude ethanol extract demonstrated that Av-EtOH was positive for the presence of phenols, steroids, terpenoids and flavonoids. However, the ethanolic extract was negative for the presence of alkaloids [13]. For the first time in the literature, our data indicate that crude ethanolic extract (Av-EtOH) obtained from the leaves of *Annona vepretorum* possess antinociceptive and anti-inflammatory profiles in different nociceptive responses generated by a chemical or thermal noxious stimulus.

The acetic acid-induced writhing test in mice has long been used as a classic model for the assessment of analgesic or anti-inflammatory properties of new agents [29]. In fact, it is a non-selective model for antinociceptive studies, since an intraperitoneal injection of acetic acid triggers the release of a variety of mediators such as substance P, bradykinins, prostaglandins especially PGI_2_ as well as pro-inflammatory cytokines such as IL-1, IL-6, IL-8 and TNF-α, stimulating the peripheral nociceptor and sensitive neurons that were responsive to the inflammatory mediators [30].

The ethanolic extract (Av-EtOH) significantly reduced the acetic acid-induced writhing in mice. The acetic acid produced 14.80 ± 2.08 writhes in the control group for 10 min after the injection. The groups previously treated with 25, 50 and 100 mg/kg of Av-EtOH exhibited a significant reduction in the number of writhings of 62, 81 and 92 %, respectively. Our results have shown that Av-EtOH possess a potent antinociceptive activity in this method.

The formalin test is an evaluation method used to measure the behavioral effectiveness of antinociceptive agents [16, 31]. The advantage of this assay over other methods of nociception is the possibility to evaluate two different types of pain over a prolonged period of time and thus allows the testing of analgesics with different mechanisms of action [30, 31]. The behavioral response to formalin followed a biphasic pattern composed of an initial acute phase (first phase), and for a longer period (second phase) and the period between phases is called the quiescent interval [32]. Drugs which act mainly centrally, such as narcotic analgesics, inhibit both phases, while peripherally acting drugs, such as indomethacin, only inhibit the late phase. Av-EtOH inhibited both phases of the formalin-induced nociception, but its effect was more prominent in the second phase.

Seeking a possible central involvement in the antinociceptive effect of Av-EtOH, hot plate and tail flick assays were conducted. The increase in latency of Av-EtOH on hot plate show provide evidences of its central effect since this is predominantly a spinal reflex and considered as central model for centrally-acting analgesic drugs [33]. This test, on thermal stimulation is associated with central neurotransmission in what the heat activates nociceptors (Aδ and C fibers) by driving the momentum of the dorsal horn of the spinal cord and subsequently to cortical centers [34].

In tail flick test, the Av-EtOH administrated at dose of 50 mg/kg (p.o) caused a significant increase in the reaction time to thermal stimuli as compared to the control group at 30 and 60 min. This test consists of a thermal stimulus, an increase in the reaction time is considered to be a parameter for evaluating central antinociceptive activity [35]. Therefore, tail flick is characterized by an acute nociception and non-inflammatory. Substances which act at central level, such as morphine, are able to suppress responses of spinal neurons to noxious thermal stimulus in the tail, increasing the latency time [36].

To confirm of the involvement of opioid receptors in the effect of the extract, naloxone (1.5 mg/kg, i.p.) was administered 30 min before administration of the extract (100 mg/kg) and morphine, being the effect of morphine and Av-EtOH reverted in the formalin and hot plate tests. As naloxone is a non-selective opioid receptor antagonist, it is suggested that this antinociceptive effect of the extract is mediated by activation of opioid receptors [37].

The inhibition on the second-phase (inflammatory nociception) of the formalin test in mice, suggested that Av-EtOH can produce antinociceptive action through the inhibition of COX and consequently prostaglandin synthesis, or other inflammatory pathway. To confirm the possible anti-inflammatory activity of Av-EtOH, the carrageenan-induced paw edema model in mice was performed.

The carrageenan-induced paw edema is an animal model widely employed for the screening of anti-inflammatory compounds or extracts and has frequently been used to assess the anti-edematogenic effect of natural products, as medicinal plants, and exhibits a high degree of reproducibility [38].

Edema formation produced by carrageenan administration in paw is a biphasic event, during 1-5 h. The initial phase (1 or 1.5 h) is predominantly a non-phagocytic edema followed by a second phase (2–5 h) with increased edema formation that remained up to 5 h [39]. Av-EtOH (25, 50 and 100 mg/kg, p.o) administered 1 h before carrageenan injection, inhibited significantly (*P* < 0.01) the increase in the edema volume at 1 and 2 h, initial phase. This phase has been induced due to the action of mediators such as histamine, serotonin and bradykinin on vascular permeability [40], which is subsequently sustained by the release of prostaglandins and nitric oxide (second-phase) with peak at 3 h. The histamine induced paw edema model was used to confirm the involvement of histamine in the anti-inflammatory effect of Av-EtOH, since the extract showed anti-inflammatory effect in the early hours of the carrageenan induced paw edema induced test. For this reason, we use the highest therapeutic dose tested in this model to evaluate if the extract would have an anti-inflammatory effect through blocking of histamine receptors [41]. Our results showed that the edema induced by histamine was significantly inhibited by Av-EtOH 100 mg/kg, p.o. in 30 min and 60 min (*P* < 0.05). Histological analysis showed less inflammation in the groups treated with the extract when the inflammation was induced by carrageenan or histamine, which demonstrates an anti-inflammatory effect and confirms the results obtained in carrageenan or histamine-induced hind paw edema in mice.

Then, the method of leukocyte migration to the peritoneal cavity was performed seeking a best characterization of the anti-inflammatory effect of Av-EtOH. The inflammation produced by this model is slow and prolonged, making it possible to evaluate the leakage of liquid, as well as cell migration, besides the participation of cytokines, enzymes, and chemical mediators, such as nitric oxide, prostaglandin E_2_, IL-1β, IL-6 and TNF-α, by utilizing different phlogistic agents [42]. These mediators are able to recruit leukocytes, such as neutrophils, in several experimental models. Av-EtOH (25, 50 and 100 mg/kg, p.o) administered 1 h before injection of carrageenan (1 %, i.p., 0.25 ml) inhibited the leukocyte migration (*P* < 0.01). A possible mechanism is an inhibition of the synthesis of many inflammatory mediators whose involvement in the cell migration is well-established.

## Conclusions

In summary, the present study has demonstrated that crude ethanolic extract of *A. vepretorum* has significant antinociceptive and anti-inflammatory properties, which are related probably with the activation of opioid receptors and inhibition of release of mediators of the inflammatory process, such as prostaglandins, histamine and neutrophils. Further research will be completed to reach the exact mechanism of action of this extract.

## References

[1] Albuquerque UP, Andrade LHC (2002). Conhecimento botânico tradicional e conservação em uma área de caatinga no estado de Pernambuco, Nordeste do Brasil. Acta Bot Bras.

[2] Albuquerque UP, Medeiros PM, Almeida ALS, Monteiro JM, Neto EMDFL, Melo JG, Santos JP (2007). Medicinal plants of the caatinga (semi-arid) vegetation of NE Brazil: a quantitative approach. J Ethnopharmacol.

[3] Almeida CFCBR, Ramos MA, Amorim ELC, Albuquerque UP (2010). A comparison of knowledge about medicinal plants for three rural communities in the semi-arid region of Northeast of Brazil. J Ethnopharmacol.

[4] Fechine IM, Silva MS, Cunha EVL, Barbosa-Filho JM, Agra MF (2002). Alcalóides isolados de *Hornschuchia obliqua* (Annonaceae). Braz J Pharmacogn.

[5] Carballo AI, Martínez AL, González-Trujano E, Pellicer F, Ventura-Martínez R, Díaz-Reval MI, López-Muñoz FJ (2010). Antinociceptive activity of *Annona diversifolia* Saff. leaf extracts and palmitone as a bioactive compound. Pharmacol Biochem Behav.

[6] Araya H (2004). Studies on annonaceous tetrahydrofuranic acetogenins from *Annona squamosa* L. seeds. B Nat Instit A E Scienc.

[7] Habib M, Waheed I (2013). Evaluation of anti-nociceptive, anti-inflammatory and antipyretic activities of *Artemisia scoparia* hydromethanolic extract. J Ethnopharmacol.

[8] Dutra LM, Costa EV, Moraes VRS, Nogueira PCL, Vendramin ME, Barison A, Prata APN (2012). Chemical constituents from the leaves of *Annona pickelii* (Annonaceae). Biochem Syst Ecol.

[9] Hamid RA, Foong CP, Ahmad Z, Hussain MK (2012). Antinociceptive and anti-ulcerogenic activities of the ethanolic extract of *Annona muricata* leaf. Braz J Pharmacogn.

[10] Foong CP, Hamid RA (2012). Evaluation of anti-inflammatory activities of ethanolic extract of *Annona muricata* leaves. Braz J Pharmacogn.

[11] Costa EV, Dutra LM, Nogueira PCL, Moraes VRS, Salvador MJ, Ribeiro LHG, Gadelha FR (2012). Essential oil from the leaves of *Annona vepretorum*: chemical composition and bioactivity. Nat Prod Commun.

[12] Dutra LM, Bomfim LM, Rocha SLA, Nepel A, Soares MBP, Barison A, Costa EV, Bezerra DP (2014). *ent*-Kaurane diterpenes from the stem bark of *Annona vepretorum* (Annonaceae) and cytotoxic evaluation. Bioorg. Med. Chem. Lett..

[13] Diniz TC, Araújo CS, Silva JC, Oliveira-Júnior RG, Lima-Saraiva SRG, Quintans-Júnior LJ, Nunes XP, Almeida JRGS (2013). Phytochemical screening and central nervous system effects of ethanolic extract of *Annona vepretorum* (Annonaceae) in mice. J. Med Plant Res.

[14] Collier HO, Dinneen LC, Johnson CA, Schneider C (1968). The abdominal constriction response and its suppression by analgesic drugs in the mouse. Brit J Pharmacol Chemother.

[15] Queiroz AC, Lira DP, Dias TL, Souza ET, Matta CB, Aquino AB, Silva LH, Silva DJ, Mella EA, Agra MF, Barbosa-Filho JM, Araújo-Júnior JX, Santos BV, Alexandre-Moreira MS (2010). The antinociceptive and anti-inflammatory activities of *Piptadenia stipulacea* Benth. (Fabaceae). J Ethnopharmacol.

[16] Hunskaar S, Hole K (1987). The formalin test in mice: dissociation between inflammatory and non-inflammatory pain. Pain.

[17] Santos DA, Fukuib MJ, Nanayakkarac NPD, Khan SI, Sousa JP, Bastos JK, Andrade SF, Silva-Filho AA, Quintão NLM (2010). Anti-inflammatory and antinociceptive effects of *Baccharis dracunculifolia* DC (Asteraceae) in different experimental models. J Ethnopharmacol.

[18] Tjolsen A, Berge OG, Hunskaar S, Rosland JH, Hole K (1992). The formalin test: an evaluation of the method. Pain.

[19] Jacob JJC, Ramabadran K (1978). Enhancement of a nociceptive reaction by opiate antagonists in mice. Br J Pharmacol.

[20] D’Amour FE, Smith DL (1941). A method for determining loss of pain sensation. J Pharm Exp Ther.

[21] Nakamoto K, Nishinaka T, Mankura M, Fujita-Hamabe W, Tokuyana S (2010). Antinociceptive effects of docosahexaenoic acid against various pain stimuli in mice. Biol Pharm Bull.

[22] Winter CA, Risley EA, Nuss GW (1962). Carrageenan-induced oedema in hind paw of rat as an assay for anti-inflammatory drugs. Proc Soc BioI Med.

[23] Huang GJ, Pan CH, Liu FC, Wu TS, Wu CH (2012). Anti-inflammatory effects of ethanolic extract of *Antrodia salmonea* in the lipopolysaccharide-stimulated RAW246.7 macrophages and the k-carrageenan-induced paw edema model. Food Chem Toxicol.

[24] Cavalher-Machado SC, Rosas EC, Brito FA, Heringe AP, Oliveira RR, Kaplan MAC, Figueiredo MR, Henriques MGMO (2008). The anti-allergic activity of the acetate fraction of *Schinus terebinthifolius* leaves in IgE induced mice paw edema and pleurisy. Int Immunopharmacol.

[25] Suleyman H, Demirezer LO, Kuruuzum A, Banoglu ZN, Goçer F, Ozbakir G, Gepdremen A (1999). Antiinflammatory effect of the aqueous extract from *Rumex patientia* L. roots. J Ethnopharmacol.

[26] Andrade GS, Guimarães AG, Santana MT, Siqueira RS, Passos LO, Machado SMF, Ribeiro AS, Sobral M, Almeida JRGS, Quintans-Junior LJ (2012). Phytochemical screening, antinociceptive and anti-inflammatory effects of the essential oil of *Myrcia pubiflora* in mice. Braz J Pharmacogn.

[27] Bastos LF, Merlo LA, Rocha LT, Coelho MM (2007). Characterization of the antinociceptive and anti-inflammatory activities of doxycycline and minocycline in different experimental models. Eur J Pharmacol.

[28] Melo MS, Guimarães AG, Santana MF, Siqueira RS, Lima ACB, Dias AS, Santos MRV, Onofre ASC, Quintans JSS, Sousa DP, Almeida JRGS, Estevam CS, Araujo BS, Quintans-Junior LJ (2011). Anti-inflammatory and redox-protective activities of citronellal. Biol Res.

[29] Mohamad AS, Akhtar MN, Zakaria ZA, Perimal EK, Khalid S, Mohd PA, Khalid MH, Israf DA, Lajis NH, Sulaiman MR (2010). Antinociceptive activity of a synthetic chalcone, flavokawin B on chemical and thermal models of nociception in mice. Eur J Pharmacol.

[30] Le Bars D, Gozariu M, Cadden SW (2001). Animal models of nociception. Pharmacol Rev.

[31] Randolph BC, Peters MA (1997). Analgesic effectiveness of ketorolac compared to meperidine in the rat formalin test. Anesth Prog.

[32] Martins MA, Bastos LC, Tonussi CR (2006). Formalin injection into knee joints of rats: pharmacologic characterization of a deep somatic nociceptive model. Pain.

[33] Nemirovsky A, Chen L, Zelman V, Jurna I (2001). The antinociceptive effect of the combination of spinal morphine with systemic morphine or uprenorphine. Anesth Analg.

[34] Pinheiro BG, Silva ASB, Souza GEP, Figueiredo JG, Cunha FQ, Lahlou S, Silva JKR, Maia JGS, Sousa PJC (2011). Chemical composition, antinociceptive and anti-inflammatory effects in rodents of the essential oil of *Peperomia serpens* (Sw.) Loud. J Ethnopharmacol.

[35] Rujjanawate C, Kanjanapothi D, Panthong A (2003). Pharmacological effect and toxicity of alkaloids from *Gelsemium elegans* Benth. J Ethnopharmacol.

[36] Fischer LG, Santos D, Serafin C, Malheiros A, Delle-Monache F, Delle-Monache G, Cechinel-Filho V, Sousa MM (2008). Further antinociceptive properties of extracts and phenolic compounds from *Plinia glomerata* (Myrtaceae) leaves. Biol Pharm Bull.

[37] Lewanowitsch T, Miller JH, Irvine RJ (2006). Reversal of morphine, methadone and heroin induced effects in mice by naloxone methiodide. Life Sci.

[38] Thomazzi SM, Silva CB, Silveira DCR, Vasconcellos CLC, Lira AF, Cambui EVF, Estevam CS, Antoniolli AR (2010). Antinociceptive and anti-inflammatory activities of *Bowdichia virgilioides* (sucupira). J Ethnopharmacol.

[39] Khan I, Nisar M, Ebad F, Nadeem S, Saeed M, Khan H (2009). Anti-inflammatory activities of Sieboldogenin from *Smilax china* Linn.: Experimental and computational studies. J Ethnopharmacol.

[40] Zhu ZZ, Ma KJ, Ran X, Zhang H, Zheng CJ, Han T, Zhang QY, Qin LP (2011). Analgesic, anti-inflammatory and antipyretic activities of the petroleum ether fraction from the ethanol extract of *Desmodium podocarpum*. J Ethnopharmacol.

[41] Muhammad N, Saeed M, Khan H (2012). Antipyretic, analgesic and anti-inflammatory activity of *Viola betonicifolia* whole plant. BMC Complem Altern Med.

[42] Loram LC, Fuller A, Fick LG, Cartmell T, Poole S, Mitchell D (2007). Cytokine profiles during carrageenan-induced inflammatory hyperalgesia in rat muscle and hind paw. Pain.

